# Structural insights into metal-metalloid glasses from mass spectrometry

**DOI:** 10.1038/s41598-020-74507-w

**Published:** 2020-10-15

**Authors:** Ananya Baksi, Soumabha Bag, Robert Kruk, Sree Harsha Nandam, Horst Hahn

**Affiliations:** 1 Institute of Nanotechnology, Karlsruhe Institute of Technology, 76344 Eggenstein-Leopoldshafen, Germany; 2 Institute of Physical Chemistry, Karlsruhe Institute of Technology, 76131 Karsruhe, Germany; 3 Herbert Gleiter Institute of Nanoscience, Nanjing University of Science and Technology, Nanjing, 210094 People’s Republic of China

**Keywords:** Characterization and analytical techniques, Mass spectrometry, Mass spectrometry, Chemical bonding, Mass spectrometry

## Abstract

Despite being studied for nearly 50 years, smallest chemically stable moieties in the metallic glass (MG) could not be found experimentally. Herein, we demonstrate a novel experimental approach based on electrochemical etching of amorphous alloys in inert solvent (acetonitrile) in the presence of a high voltage (1 kV) followed by detection of the ions using electrolytic spray ionization mass spectrometry (ESI MS). The experiment shows stable signals corresponding to Pd, PdSi and PdSi_2_ ions, which emerges due to the electrochemical etching of the Pd_80_Si_20_ metallic glass electrode. These fragments are observed from the controlled dissolution of the Pd_80_Si_20_ melt-spun ribbon (MSR) electrode. Annealed electrode releases different fragments in the same experimental condition. These specific species are expected to be the smallest and most stable chemical units from the metallic glass which survived the chemical dissolution and complexation (with acetonitrile) process. Theoretically, these units can be produced from the cluster based models for the MG. Similar treatment on Pd_40_Ni_40_P_20_ MSR resulted several complex peaks consisting of Pd, Ni and P in various combinations suggesting this can be adopted for any metal-metalloid glass.

## Introduction

Most structural, physical and chemical properties of polycrystalline alloys depend directly on the crystallographic structure, characterized by long range order, and the microstructure, i.e., the ensemble of all defects within the material. The microstructure of alloys can be tailored by means of the processing steps resulting in control of properties. In contrast, the amorphous structure of metallic glasses with similar compositions that exhibit only short- and medium range order results in maze-like pattern in high resolution transmission electron microscopy (TEM) without any discernible microstructural information^1–3^. In view of their distinct physical and chemical properties, metallic glasses have gained popularity in several scientific disciplines. Advanced synthesis and processing methods have been employed to modify the local structure of these glassy systems by the controlled introduction of defects, such as interfaces and precipitates^4–14^. The absence of long range ordering in metallic glasses complicates the understanding of their local structure^3^. The smallest building blocks (structural local motifs or clusters) and the direct chemical bonding between the constituents have been determined in several experiments and predicted by theoretical models. Information on the bond length as well as nearest and next-nearest neighborhood has been provided by a variety of experiments^15–18^. Current research on the structure of metallic glasses has revealed favored and unfavoured local atomic packing (structural motifs) and a significant diversity in the short-range ordering of metallic glasses^14^.

Several structural models have been introduced to describe the local structure and to understand the origin of the unusual properties of metallic glasses compared to their crystalline counterparts^4,10^. In addition to long studied dense random packing of hard spheres and ‘solid-like’ and ‘liquid-like’ regions in MGs, cluster-based local ordering is a widely accepted model for structurally disordered, yet macroscopically homogeneous systems^11,19^. Tailoring the population of local structural motifs (or building blocks) in a controllable manner may help to improve and engineer selective properties of metallic glasses. Hence deciphering the structural dissimilarity among the local motifs is necessary to establish a causal link between the key local structures and macroscopic properties^2,3,14,20,21^. Experimentally, the process is far more challenging as it requires high-end instrumental facility such as aberration corrected transmission electron microscopy (TEM) or synchrotron radiation sources^16^.

The understanding of the unusual mechanical/magnetic properties and the manipulation of the structure to optimize and improve properties of metallic glasses is directly related at the atomic level to the chemical bonding between the constituent elements^15,20,22^.

In search of the most stable configuration in metal-metalloid type metallic glasses, we introduce a novel method to visualize the chemical building blocks of single phase complex amorphous materials using a combination of easily accessible mass spectrometry (MS) additionally supported by Raman spectroscopy. The metallic glasses are de-alloyed or dissolved by electrochemical corrosion. In a process known as “Electrolytic Spray”, which is a fairly recent technique, introduced and described by Cooks and co-workers, electrospray ionization is employed to form ions for further characterization^23,24^. We have adopted this technique to determine the immediate local chemical bonds among the constituents of a metallic glass. It is the principal idea of the experiment that the strongest bonds between the constituent elements will remain stable and can be considered as the elemental building blocks of the metallic glass, stabilizing the amorphous structure. The combination of two techniques (mass spectrometry and Raman spectroscopy) can be employed to determine the bonding and the active constituents, which are responsible for the stabilization of the amorphous phase in the model systems Pd_80_Si_20_ and Pd_40_Ni_40_P_20_.

## Results and discussion

### Electrolytic spray ionization mass spectrometry

**Figure 1:**
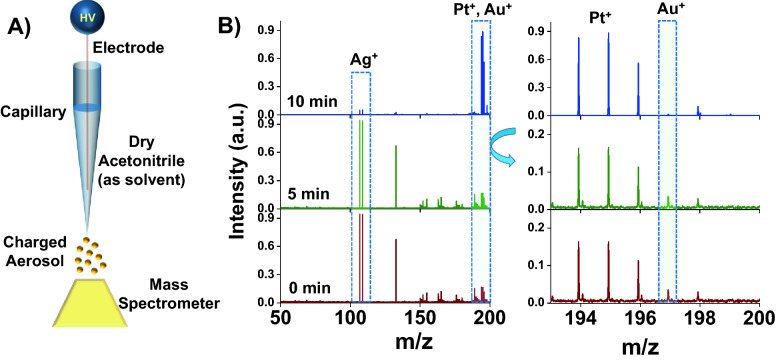
Experimental setup and proof of concept. **(A)** The electrolytic spray setup is illustrated. **(B)** Electrolytic spray of 99.99% pure Pt-wire (rest are Au and Ag impurities) in positive ion mode showing Pt^+^ as major ion. During the initial spraying time more reactive impurities like Ag and Au were detected due to electrochemical etching.

In electrolytic spray ionization mass spectrometry, metals are electrochemically etched with acetonitrile (ACN) in the presence of a high voltage. The solvated metal ions are directed to a mass spectrometer for analysis. Up to now, the etched ions have been deposited in desired shapes on preferred substrates in the form of nanoparticles as described by Cooks et al*.*^23,24^ in the past and Spolenak et al*.* more recently^25^. In both cases, the sacrificial metal electrode acts as an anode. In the current work, the electrolytic spray process has been used in a completely different perspective where a MSR was used as an electrode. Electrochemical reaction between the MSR and ACN produced solvated ions in the spray plume which were subsequently detected using a Waters Synapt G2S mass spectrometer. Electrolytic spray was achieved and calibrated by modifying an existing nano-electrospray ionization (NESI) setup as shown in Fig. 1A.

### Electrochemical etching and dealloying from polycrystalline metal alloys

When a pure polycrystalline metal M was used, only M^+^ and M(CH_3_CN)_n_^+^ and a few M(H_2_O)^+^ species were observed. This was observed previously as the pure metal can have only one type of metal–metal bonding (see Fig. S1 for polycrystalline Cu)^23,24^. A Pt-wire (99.99% purity) was used as an electrode to optimize the experimental parameters. Close to 1 kV voltage was applied to generate a stable ion current during the electrolytic spray. During initial spray period, a strong signal at m/z 107 and 109 was observed along with a peak envelope at m/z 194–198. The peaks were assigned as Ag^+^ and Pt^+^. Upon careful examination, a peak for Au^+^ at m/z 197 was also found. Ag and Au are present as common impurities in the commercial Pt-wire and are etched out first as those are more reactive than Pt. The intensity of Ag^+^ and Au^+^ decreased with time (0–10 min) as the impurity was removed from the electrode as per their electrochemical reactivity and consequently, the Pt^+^ signal became stronger. The data are presented in Fig. 1B.

This is an example of dealloying during electrolytic spray^26^ and demonstrates the sensitivity of the process. It should be noted that non-reactive organic solvent acetonitrile was used for electrochemical etching here instead of strong acid like hydrofluoric acid, sulphuric acid etc. (or alkali in some cases). 

### Building blocks of Pd_80_Si_20_ MSR

**Figure 2:**
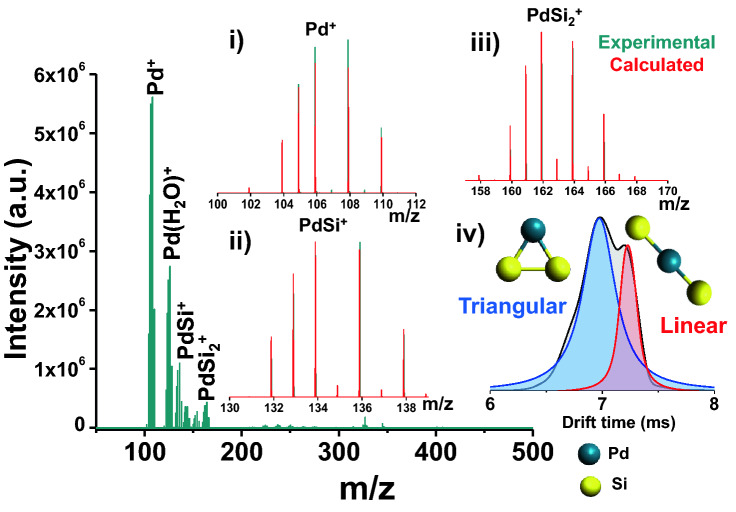
Electrolytic spray MS using Pd_80_Si_20_ MSR. Representative electrolytic spray (in NESI MS) of Pd_80_Si_20_ MSR in positive ion mode showing different Pd_n_Si_m_ ions. Expanded mass regions for each ion species are shown in inset (i)–(iii). Extracted ion mobilogram of PdSi_2_^+^ is shown in (iv) and possible structures of the ions are shown alongside the peaks.

Armed with the idea, we have selected a well-studied single phase glassy Pd_80_Si_20_ melt-spun ribbon (MSR)^27–29^ as an electrode and followed the above mentioned process. It should be noted here that electrochemical etching from any metallic glass using ACN has not been attempted before. The data are shown in Fig. 2 [corresponding X-ray diffraction (XRD) in Fig. S2]. During electrolytic etching using NESI MS, the main constituents detected in the mass spectra are Pd^+^ (m/z 102–110), PdSi^+^ (m/z 132–138) and PdSi_2_^+^ (m/z 158–168) with a few water adducts in the positive ion mode. This observation indicate that electrochemical etching is happening without selectively following the electrochemical reactivity of Pd and Si. Simultaneous release of Pd^+^, PdSi^+^ and PdSi_2_^+^, all of which has different reactivity, is possible only if they are part of the same microscopic unit in the Pd_80_Si_20_ MSR alloy.

Considering the dissolution process of the alloy, these species can be considered as the active chemical building blocks in the Pd_80_Si_20_ amorphous phase and, thus, are the smallest stable and predominant entities. The entity PdSi has been predicted from measurements of the radial distribution function (RDF) on the same system but the existence of PdSi_2_^+^ was not known^28–31^. The ratio of Pd and Si in the MSR was found to be ~ 3.8:1 from mass spectrometric intensity, which is very close to the actual ratio 4:1. In order to ensure that the reaction is not just a reflection of the surface activity of the MSR, more than 30 h of experiment was performed on randomly selected days with the same experimental parameters using the same MSR (in triplicate). In all cases, similar intensity ratios among the detected species were found. Additionally, the triatomic PdSi_2_^+^ species can possess different structures (linear or triangular), which can be separated using ion mobility (IM) coupled with regular MS. When these ions were allowed to pass through the ion mobility drift cell, PdSi_2_^+^ showed two isomeric peaks in the extracted ion mobilogram indicating two different types of PdSi_2_^+^ geometry. Ion geometries were optimized using density functional theory (DFT) with TPSS functional and def2-TZVPP basis set as implemented in the TURBOMOLE package. The triangular structure (77% population from IM-MS) is found to be more stable compared to the linear one (23% population from IM-MS) which was further confirmed by IM-MS.

### Identification of the structural motifs

**Figure 3:**
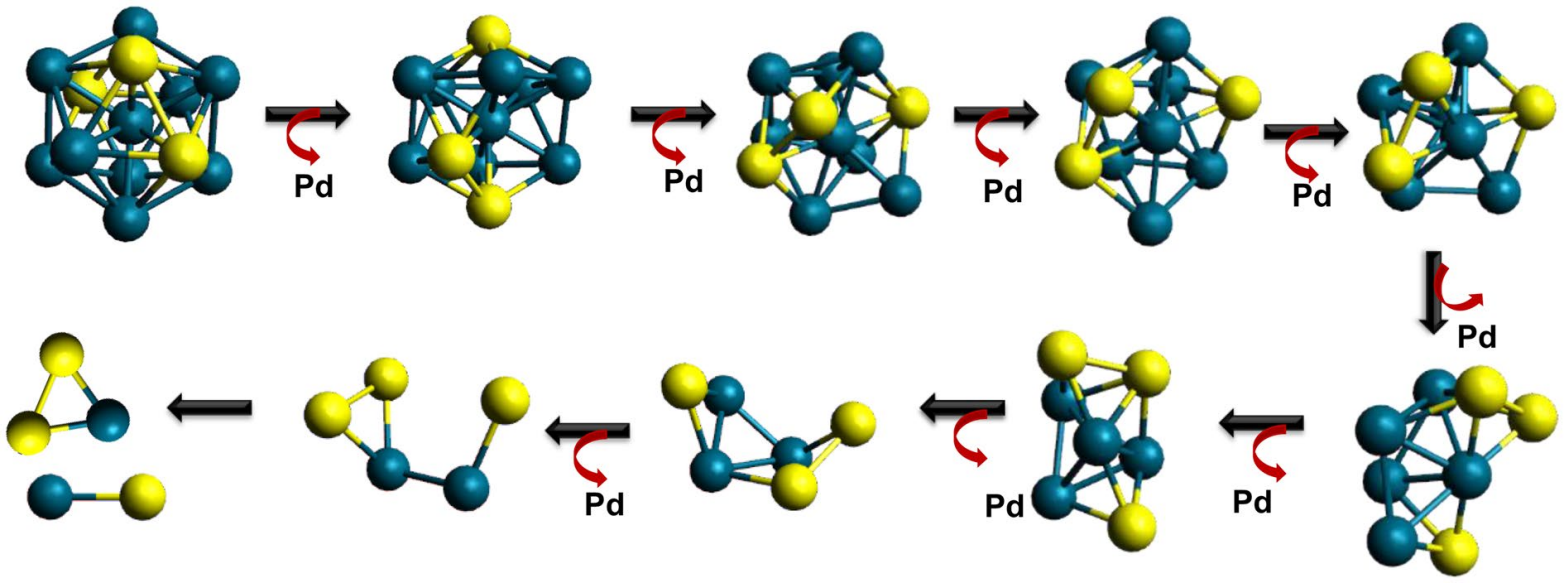
Mechanism of Pd, PdSi and PdSi_2_ ion formation in the atomic scale. Possible fragmentation sequence in the atomic scale in the presence of ACN. Pd_10_Si_3_ icosahedron cluster is selected here to represent Pd_80_Si_20_ MSR microstructure. Note that all these steps can happen simultaneously. The current mechanism do not claim the existence of the specific Pd_10_Si_3_ cluster as the only possible structure. However, despite of the cluster nuclearity and geometry, these three stable species can be seen in any cluster containing both Pd (blue sphere) and Si (yellow sphere).

The existence of these particular species can be understood following the cluster based models generally used for the metallic glass. A plausible electrochemical fragmentation mechanism involving a Pd_10_Si_3_ (microscopic part of Pd_80_Si_20_ MSR) icosahedron cluster is shown for clarity (Fig. 3). The icosahedron geometry is a suggested local structural model for metallic glass and was chosen to represent 4:1 overall atomic ratio. While non-icosahedron solute centered quasi-equivalent coordination polyhedra is predicted for such Pd-Si system^32,33^, a simpler icosahedron model is used here to understand the possible pathway which can lead to PdSi_2_^+^ as seen in the current experimental condition shown in Fig. 2. Existence of the moieties (PdSi and PdSi_2_) in the Pd_80_Si_20_ MSR is additionally confirmed by Raman spectroscopy (see later).

### Raman spectroscopy

Apart from mass spectrometry, Raman spectroscopy can help in understanding the materials. The optical phonon (Γ_O_) signal at 520 cm^−1^ of pure crystalline Si is generally used as a standard for Raman spectroscopy. Raman spectra in the range of 200–550 cm^−1^ are shown in Fig. S3. Amorphous Si shows a broad hump at ~ 480 cm^−1^. When the amorphous and crystalline (see later for the preparation) Pd_80_Si_20_ alloys were analyzed by Raman spectroscopy, none of the samples showed any peak at 520 cm^−1^ or hump around ~ 480 cm^−1^. This confirms the absence of amorphous and crystalline Si in the samples. This is in agreement with the previous RDF calculation which predicted no long range Si–Si bonding. Two peaks at ~ 190 cm^−1^ and ~ 210 cm^−1^ in the amorphous specimen are due to PdSi type of structure. Other peaks at 280, 332, 358 cm^1^ are attributed to the disordered PdSi while diffused and broad peaks in the range of 200–450 cm^−1^ are due to disordered PdSi_2_. No sharp signal comprising of pure PdSi or PdSi_2_ were observed, which can be understood to disorder induce Raman scattering from the amorphous alloy^34–39^. Results of the Raman spectroscopy confirm the presence of PdSi and PdSi_2_ in the Pd_80_Si_20_ MSR sample. These components are also seen during NESI MS measurement. Raman spectroscopy has been implemented here as a complementary method to the NESI MS.

### Difference between amorphous and crystalline phase

Amorphous to crystalline phase transformation has been carried out upon heating the amorphous material well above its crystallization temperature. Previously reported results show that the crystallization temperature of Pd_80_Si_20_ MSR is 648 K where pure Pd is formed in majority along with Pd_3_Si. To obtain a fully crystallized sample, the MSR was heated at 673 K for 3 h in vacuum and the annealed ribbon was subsequently analyzed.

Mass spectra arising from the annealed ribbon visibly differ from that of amorphous analogue. It shows only the characteristic feature of Pd ions with H_2_O and CH_3_CN complexes of Pd. No signal for PdSi or PdSi_2_ were found after annealing (Fig. S4). The other component Pd_3_Si was not seen which is probably inert to such electrochemical treatment and hence was not etched out. The other reason is surface segregation of Pd after annealing leading to dealloying as discussed in Fig. 1. Presence of nanosized Pd was further confirmed by surface enhanced Raman spectroscopy (SERS) (see Note 1 and Fig. S5 in supplementary information for details). Raman spectroscopy also could not identify Pd_3_Si at the surface of the annealed ribbon.

### Building blocks of Pd_40_Ni_40_P_20_ MSR

**Figure 4:**
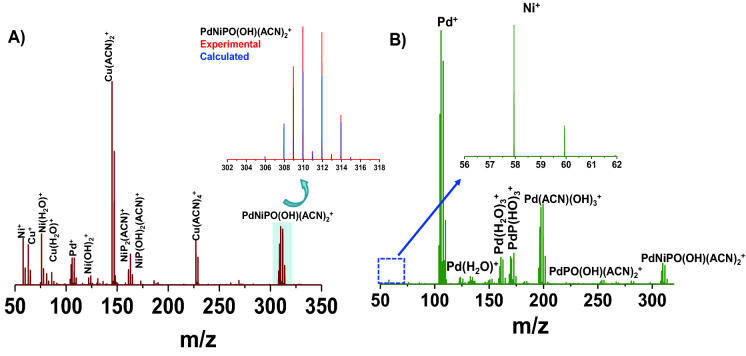
Electrolytic spray MS using Pd_40_Ni_40_P_20_ MSR. **(A)** Electrolytic spray using an amorphous Pd_40_Ni_40_P_20_ MSR is showing peaks of Ni, Pd and P at various composition in the positive ion mode. Crystalline Cu is present as impurity on the surface and hence been electrolytically etched. Complex peak at m/z 309.8 corresponds to PdNiPO(OH)(ACN)_2_ which is expanded in the inset and compared with calculated isotope pattern. **(B)** Tandem Mass spectrometry (MS/MS) of m/z 309.8 at laboratory collision energy 25 V showing Ni and ACN loss and finally resulting in Pd^+^ at higher collision energy. Presence of Ni is also seen at lower mass (expanded in the inset). Collision energy dependent MS/MS spectra are shown in Fig. S6.

To extend the feasibility of the process to differentiate other amorphous systems, the ternary glassy alloy Pd_40_Ni_40_P_20_ was analyzed following identical methods as discussed above. This glass has also been studied extensively^40–45^. In this case, the material is composed of Pd–Ni metallic bonds and both Pd-P and Ni–P covalent bonds as reported in the previous studies. When the amorphous Pd_40_Ni_40_P_20_ ribbon was subjected to electrolytic spray at 1 kV potential (NESI MS), multiple pure Ni, Pd and a few water and ACN adducts were seen (see Fig. 4). The small quantity of crystalline Cu present on the surface is an impurity in the system being etched simultaneously. The presence of Cu arises most likely from the synthesis, when the molten mixture of Ni_2_P and Pd is collected on a water-cooled Cu surface. The Ni–P type bonds in the metallic glass are confirmed from the NiP_2_ and NiP containing ions in the mass spectrum. A strong and stable signal was found at m/z 309.8 which is assigned as PdNiPO(OH)(ACN)_2_^+^. Presence of these ion was further confirmed by tandem mass spectrometry (MS/MS) where Ni and solvent loss peaks were observed (Fig. S6). Initial loss of Ni was observed and the resulting species PdPO(OH)(ACN)_2_^+^ was seen at m/z 251.9. With increasing collision energy (0 eV to 30 eV, laboratory collision energy), other components were also lost and at the highest collision energy, only signal of Pd^+^ fragment was detected. After careful examination, the peak due to Ni^+^ was also seen (inset of Fig. 4B and Fig. S6B) in low mass region but the intensity was far lower compared to Pd^+^ intensity. Probably the most favored fragmentation pathway (in MS/MS) is by neutral Ni loss and hence Ni^+^ is not seen at higher intensity. The Pd–Ni unit was not seen in the current MS process but due to the presence of P in the system species containing both Pd and Ni with P were observed.

After the NESI MS analysis, the MSR was heated at 693 K in vacuum for 2 h above its reported glass transition temperature (575 K) and the crystalline alloy was analyzed again using NESI MS. The mass spectral signal from the crystalline sample is completely different from the amorphous one. The strong signal at m/z 148.5 was assigned at Ni_2_P, which is known to be a stable phase in the crystalline alloy and preformed Ni_2_P was used as Ni and P source during the synthesis of the glass (Fig. S7). Previous reports suggest the existence of Ni_3_P, which was not observed in the present experimental condition. After crystallization, Pd–P bonds were not observed and only free Pd^+^ with a few ACN and water adducts were found in the mass spectrum. Apart from free Ni and Pd, Ni_2_Pd_2_P phase is also known for the crystalline phase, absence of which can be explained by similar metallic bond breaking during electrospray analysis.

## Conclusions

A direct method, based on electrolytic spray ionization mass spectrometry and Raman spectroscopy, to understand the most stable structural components and local bonding in amorphous materials namely, in Pd_80_Si_20_ and Pd_40_Ni_40_P_20_ metallic glasses is presented here. An inert solvent, acetonitrile was used for electrochemical etching during mass spectrometric experiment and subsequently for carrying the ions to the mass spectrometer. While electrochemical dealloying is evident for polycrystalline metals, single phase glassy alloys showed distinct ions with various combination of the constituent elements. Amorphous Pd_80_Si_20_ MSR showed signals for Pd, PdSi and PdSi_2_ ions, which are further supported by Raman spectroscopy. After crystallization, only Pd containing ions were seen in positive ion mode. Absence of other entities in the crystalline sample could be understood from the surface segregation of nano-Pd on the surface as seen from Raman spectroscopy. A simple cluster based structural model has been used to understand the most stable building blocks for such materials. Irrespective of the nuclearity and geometry of the clusters, only these three species (for Pd_80_Si_20_) are possible as the smallest stable building blocks which are now experimentally proven. A similar study on Pd_40_Ni_40_P_20_ glass showed characteristic peaks containing Ni–P and Pd–P in different complex combination. Understanding the difference of the local structure of different forms of amorphous materials with similar chemical composition would allow to tune their physical properties such as high mechanical strength, enhanced magnetism etc. Detailed theoretical calculations of the proposed phases and their three dimensional projection in voxel by voxel fashion will reveal more about the chemical structure of the materials.

## Methods

### Materials

#### Synthesis of Pd_80_Si_20_ and Pd_40_Ni_40_P_20_ MSR

Both the glasses were synthesized following conventional melt quenching technique and was spun on a rotating Cu disc. For Pd_40_Ni_40_P_20,_ preformed Ni_2_P was used as Ni and P source.

### Mass spectrometry

All mass spectrometric studies were performed in positive ion nano-electrospray ionization (NESI) mode in a Waters’ Synapt G2S high definition mass spectrometer (MS) equipped with travelling wave ion mobility (IM) separation (TWIMS) and time of flight mass analyzer. About 1 kV DC high voltage was applied on the borosilicate glass capillary containing the metal/glassy alloy electrode and pure acetonitrile (ACN) from Sigma Aldrich was used as spray solvent. Cone voltage and source offset were set at 20 and 30 V, respectively. For ion mobility separation, IMS velocity and wave height were set at 650 m/s and 40 V, respectively.

### Raman spectroscopy

All Raman spectroscopic measurements were performed using a Renishaw in via confocal Raman spectrometer equipped with 532 and 785 nm laser with maximum laser power of 50 mW. Most of the measurements were performed with 532 nm laser. Only SERS study was performed with 785 nm laser to avoid emission from the analyte.

### DFT calculation

Structure of the isomeric PdSi_2_ ions were optimized using density functional theory (DFT) with TPSS functional and def2-TZVPP basis set as implemented in the TURBOMOLE package. Coordinates of the optimized triangular and linear PdSi_2_^+^ structures are in supplementary information.

## Supplementary information

Supplementary Information.
