# Supplementary material for: Structural insights into metal-metalloid glasses from mass spectrometry
Source: Sci Rep. 2020 Oct 15;10:17467. doi: 10.1038/s41598-020-74507-w (PMC7567878; doi:10.1038/s41598-020-74507-w)
Supplement: Supplementary file 1 — Supplementary Information. [file 41598_2020_74507_MOESM1_ESM.pdf]

Supplementary Information

Structural Insights into Metal-Metalloid Glasses from Mass Spectrometry

Ananya Baksi,[1,2†*] Soumabha Bag,[1,2†*] Robert Kruk,[1] Sree Harsha Nandam,[1] and Horst Hahn[1,3*]

[1]*Institute of Nanotechnology, Karlsruhe Institute of Technology, 76344 Eggenstein-Leopoldshafen, Germany*
[2]*Institute of Physical Chemistry, Karlsruhe Institute of Technology, 76131 Karsruhe, Germany*
[3]*Herbert Gleiter Institute of Nanoscience, Nanjing University of Science and Technology, Nanjing 210094, P. R. China*

[*]Email: ananya.baksi@kit.edu, soumabha.bag@kit.edu and horst.hahn@kit.edu

[†]These authors have contributed equally

Content	Description	Page

Fig. S1	Electrolytic spray from crystalline Cu wire	Page S3
Fig. S2	XRD of $Pd_{80}Si_{20}$ MSR	Page S4
Fig. S3	Comparison between amorphous and crystalline phase (MS)	Page S5
Note 1	Raman Spectroscopy	Page S4
Fig. S4	Comparison between amorphous and crystalline phase (Raman Spectroscopy)	Page S7
Fig. S5	SERS using crystalline $Pd_{80}Si_{20}$	Page S8
Fig. S6	Tandem mass spectrometry (MS/MS)	Page S9
Fig. S7	Electrolytic spray with annealed $Pd_{40}Ni_{40}P_{20}$	Page S9
Coordinates of DFT optimized isomeric $PdSi_2^+$		Page S10

Supplementary Information 1

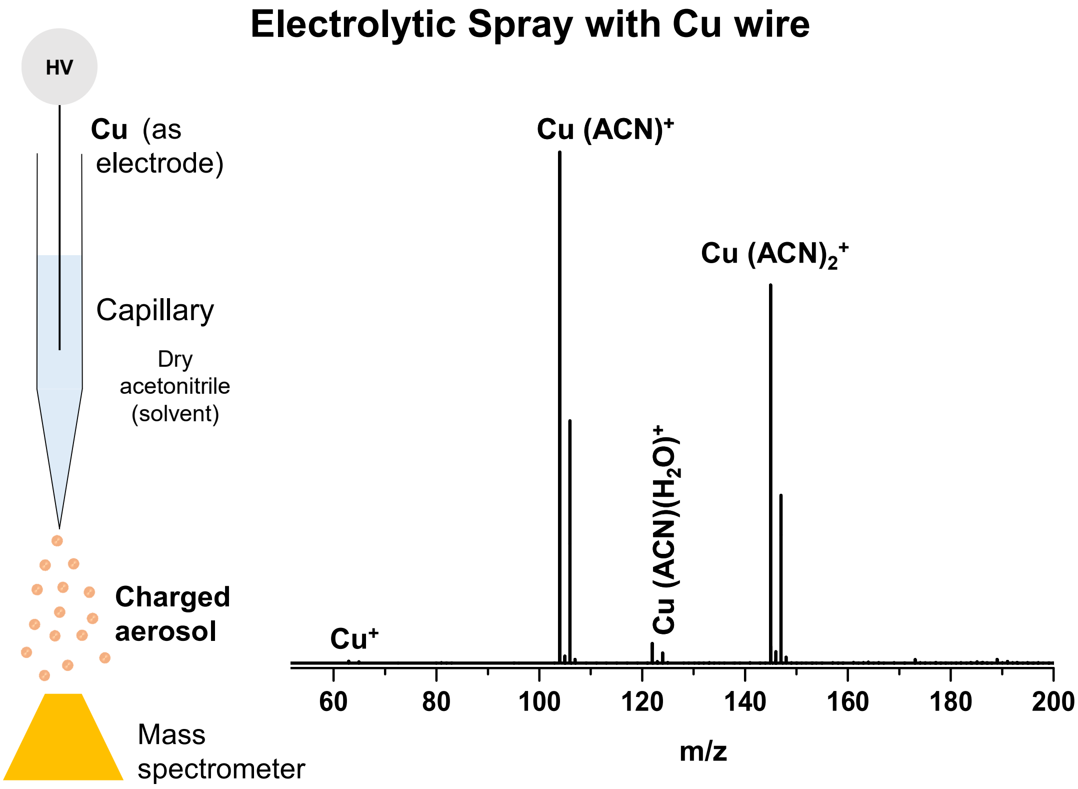

Fig. S1. Electrolytic spray MS using a crystalline Cu wire. Electrolytic spray MS using a Cu wire is showing peaks of Cu and ACN adducts in the positive ion mode. Experimental setup is schematically shown on the left panel.

Supplementary Information 2

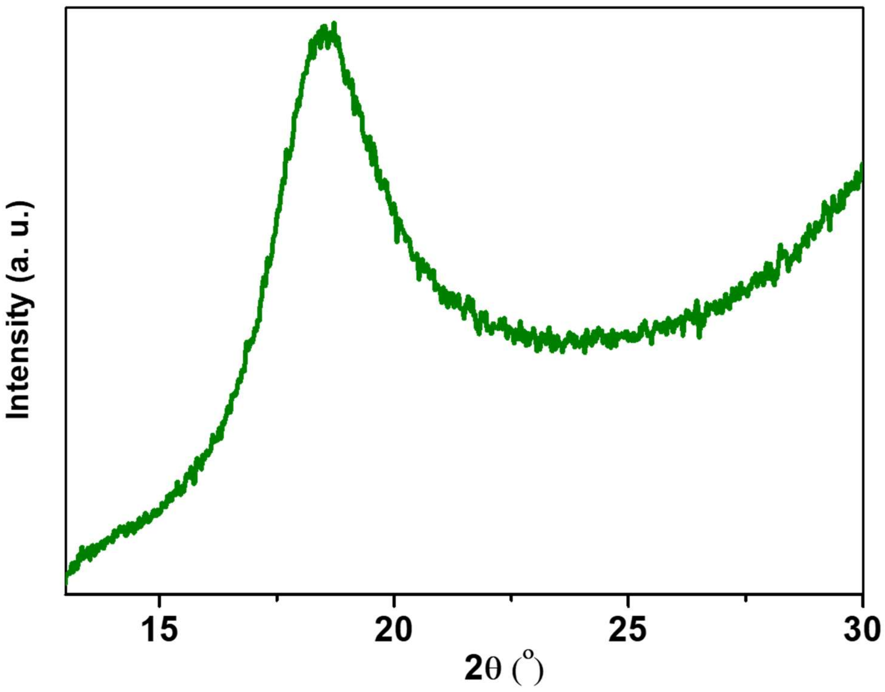

Fig. S2. XRD of amorphous $Pd_{80}Si_{20}$. XRD of $Pd_{80}Si_{20}$ (measured with Mo Kα source) MSR is showing characteristic *halo* peak for glassy alloys.

Supplementary Information 3

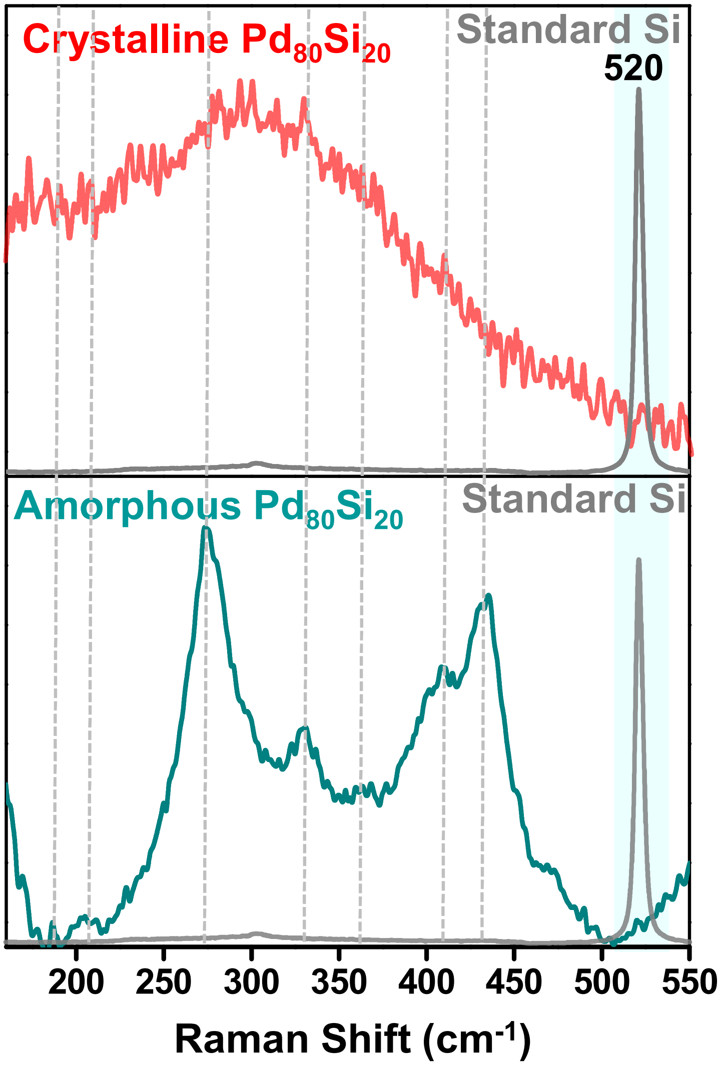

Fig. S3. Distinguishing amorphous and annealed phase by Raman Spectroscopy. Raman spectra of amorphous and annealed $Pd_{80}Si_{20}$ MSR showing distinctly different peaks. While amorphous MSR is showing characteristic peaks for PdSi and $PdSi_2$ phase, annealed MSR do not show any of the peaks. Both the spectra are compared with standard Si wafer (for clarity) showing characteristic crystalline Si peak at 520 cm^{-1}.

Note 1. Raman Spectroscopy

We have studied amorphous and annealed $Pd_{80}Si_{20}$ separately using Raman Spectroscopy. At similar experimental condition, crystalline MSR did not show any characteristic peaks of PdSi, $PdSi_2$ or Pd_3Si, further confirming these phases were not present at the surface of heat-treated MSR. Raman spectroscopy was unable to offer additional information on chemical bonding from annealed $Pd_{80}Si_{20}$ surface. However, surface enhanced Raman spectroscopy (SERS) study where Rhodamin 6G (R6G) was used as an analyte (Fig. S5) confirmed the presence of excess nanosized Pd at the surface. In order to avoid fluorescence background from R6G, 785 nm laser

was used as excitation source during SERS measurement. Scattering intensity at 1363 cm^{-1} was used for enhancement factor (EF) calculation. Although nano-Pd is not a promising as SERS substrate, as low as 1 μM of R6G could be detected on the annealed $Pd_{80}Si_{20}$ melt-spun ribbon surface with EF of 10^4 which is still high considering SERS activity of Pd nanoparticles reported so far. However, the SERS result confirm the presence of metallic Pd which seggrate at the surface following the heating experiment of the $Pd_{80}Si_{20}$ MSR sample. Analogous SERS study on amorphous MSR did not show any enhancement confirming homogeneity of the sample and further confirm no nano-Pd segregation on surface and hence no SERS activity was observed.

Supplementary Information 4

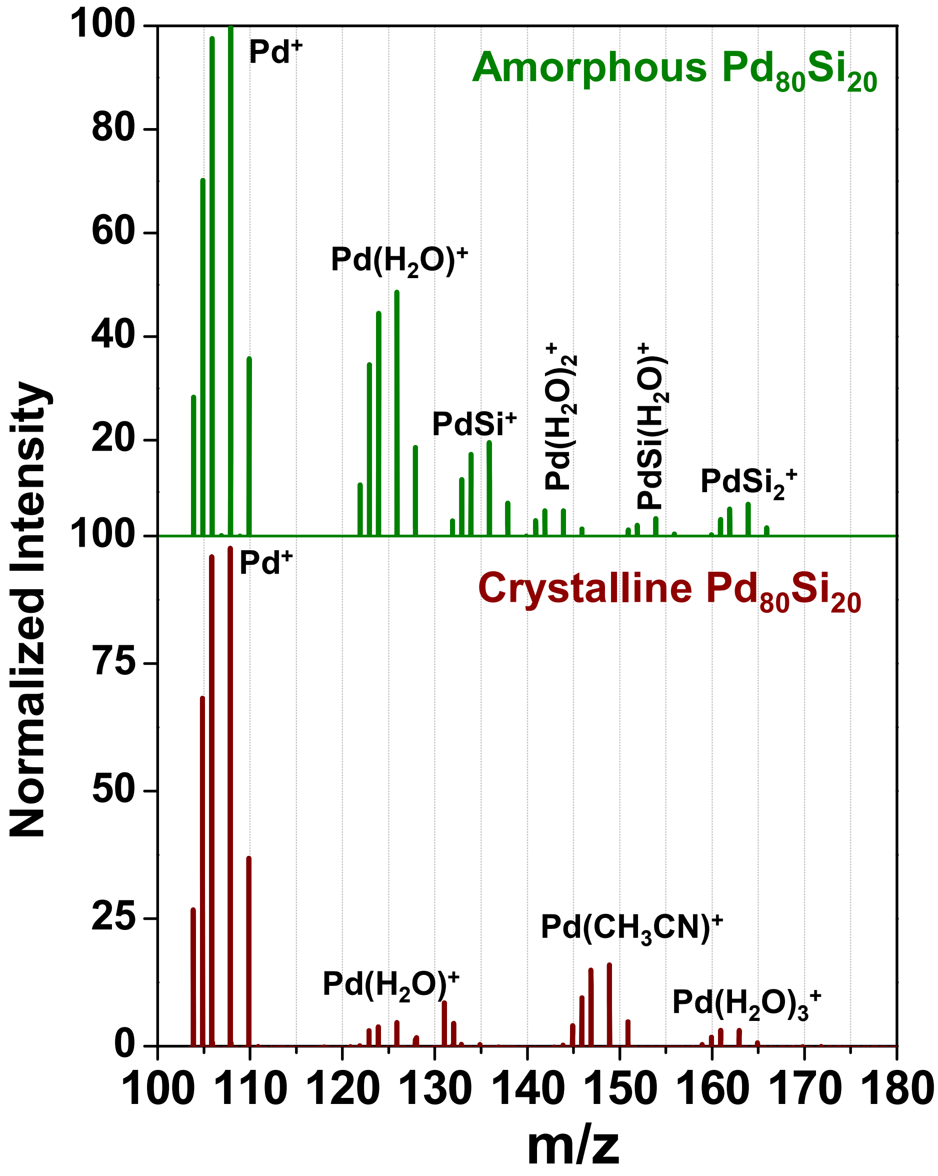

Fig. S4. Distinguishing amorphous and crystalline phase by MS. Comparative electrolytic spray ionization mass spectra of amorphous and crystalline $Pd_{80}Si_{20}$ showing disappearance of PdSi and $PdSi_2$ ions upon crystallization.

Supplementary Information 5

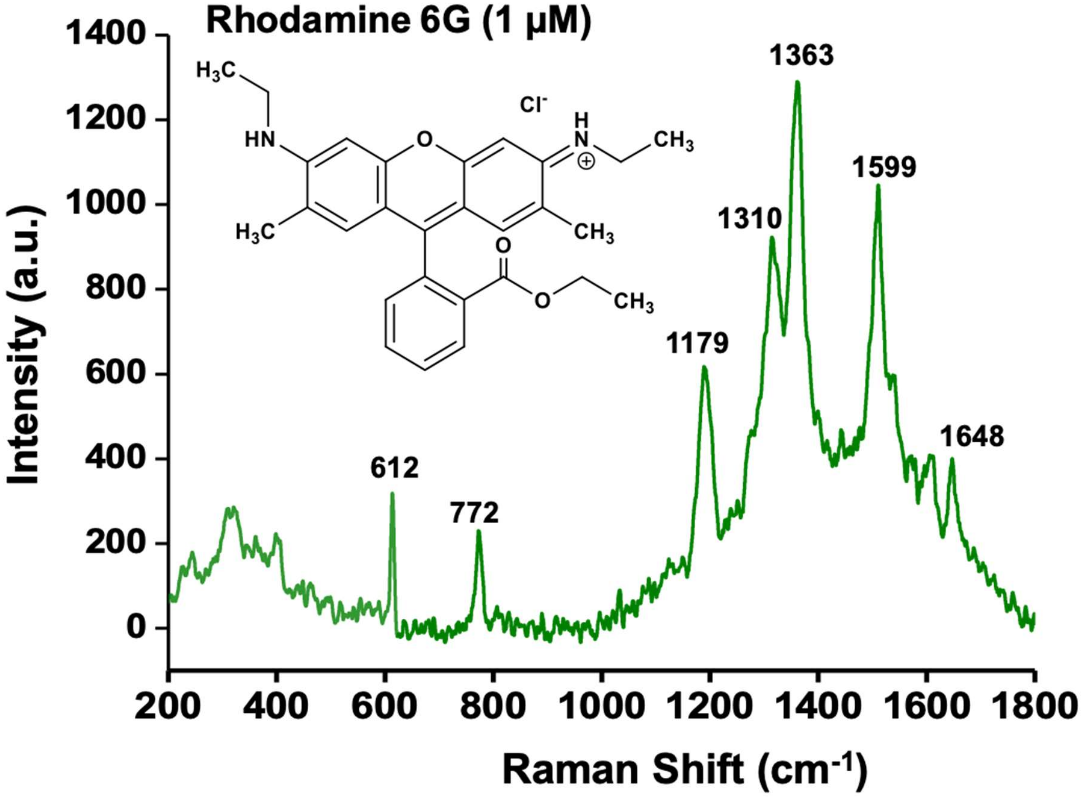

Fig. S5. SERS activity of annealed $Pd_{80}Si_{20}$. SERS spectrum of 1 μM Rhodamine 6G on annealed $Pd_{80}Si_{20}$ surface showing all characteristic peaks. Enhancement factor was calculated using intensity of 1363 cm^{-1} peak which was found to be 10^4.

Supplementary Information 6

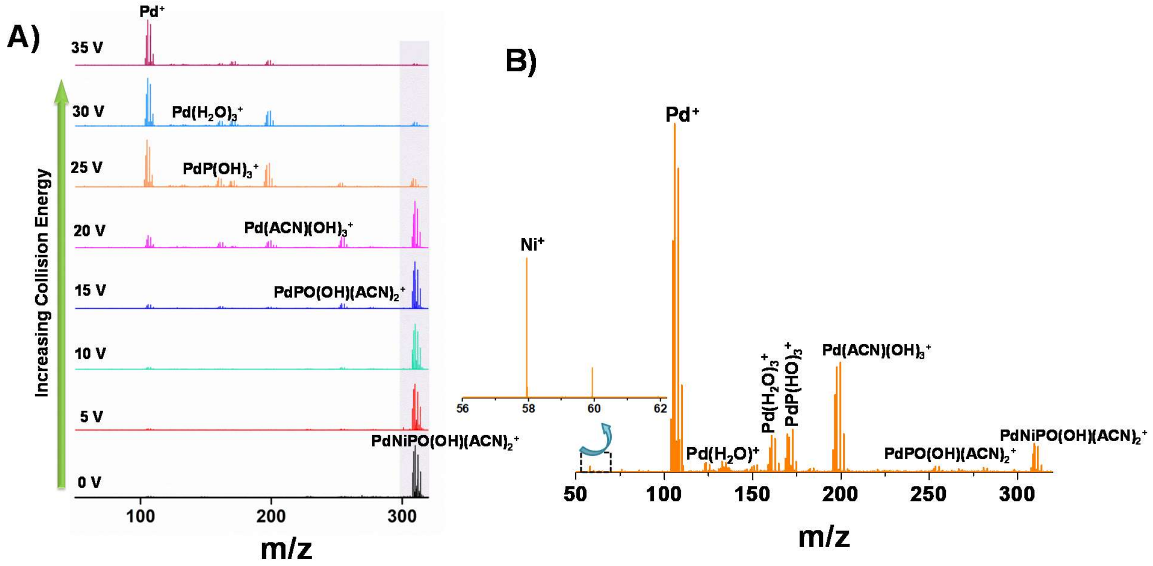

Fig. S6. Tandem mass spectrometry. A Tandem Mass spectrometry (MS/MS) of m/z 309.8 at varying collision energy showing Ni and ACN loss and finally resulting in Pd^+ at higher collision energy. Collision energy (laboratory) was increased from 0 eV to 35 eV with a step of 5 eV. Expanded spectra at CE 25 eV is shown in **B** for clarity. Peak of Ni was also seen at low mass region.

Supplementary Information 7

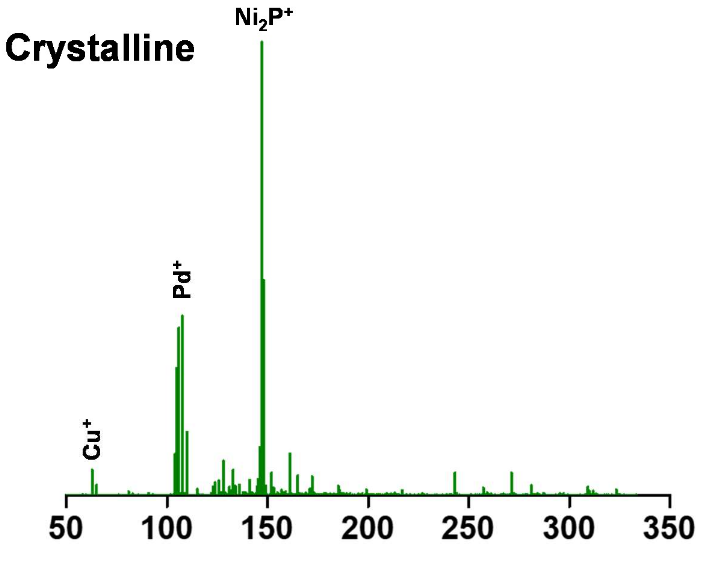

Fig. S7. Identifying active component in annealed $Pd_{40}Ni_{40}P_{20}$. Electrolytic spray MS (NESI MS) using an annealed $Pd_{40}Ni_{40}P_{20}$ ribbon is showing peaks comprised of most stable Ni_2P^+ and Pd^+. Other inter-metallic peaks were not found in the present study.

Supplementary Information 8

Coordinates of DFT optimized isomeric $PdSi_2^+$

Triangular

Atom	x	y	z
Pd	0.00000	0.00000	1.27897
Si	1.19835	0.00000	-0.63998
Si	-1.19835	0.00000	-0.63998

Linear

Atom	x	y	z
Pd	0.00000	0.00000	0.03899
Si	0.00000	0.00000	2.23699
Si	0.00000	0.00000	-2.27598
